# Patterns and predictors of fall injury transitions among Korean older adult fallers: a 2-year longitudinal study

**DOI:** 10.1038/s41598-022-26665-2

**Published:** 2022-12-23

**Authors:** Gwang Suk Kim, Mi-So Shim, Chang Won Won, Miji Kim, Seoyoon Lee, Namhee Kim, Min Kyung Park

**Affiliations:** 1 Mo-Im Kim Nursing Research Institute, College of Nursing, Yonsei University, Seoul, Republic of Korea; 2 College of Nursing, Keimyung University, Daegu, Republic of Korea; 3 Department of Family Medicine, College of Medicine, Elderly Frailty Research Center, Kyung Hee University, Seoul, Republic of Korea; 4 Department of Biomedical Science and Technology, East-West Medical Research Institute, College of Medicine, Kyung Hee University, Seoul, Korea; 5 Interdisciplinary Graduate Program in Social Welfare Policy, Yonsei University, Seoul, Republic of Korea; 6 College of Nursing Brain Korea 21 FOUR Project, Yonsei University, Seoul, Republic of Korea; 7 Department of Nursing, Graduate School of Yonsei University, Seoul, Republic of Korea

**Keywords:** Geriatrics, Health services, Public health

## Abstract

This study was conducted to identify fall injury patterns, the transition from the baseline to follow-up, and the factors associated with the identified fall injury patterns using data obtained from the Korean Frailty and Aging Cohort Study. The participants were 566 community-dwelling older adults with fall experience. Three fall injury patterns were identified as the baseline and follow-up periods. The probability that the participant in the “fracture injury” pattern at Time 1 transitioned to the “fracture injury” pattern at Time 2 was 0.098. The factors associated with the “bruising and/or sprain injury” pattern were education level (relative risk ratio [RRR] = 0.55, p = 0.012), alcohol consumption (RRR = 0.50, p = 0.034), and balancing in tandem position (RRR = 2.77, p < 0.001). In the “fracture injury” pattern, male (RRR = 0.22, p = 0.038), frailty score (RRR = 0.58, p = 0.042), “bruising injury” (RRR = 0.23, p = 0.007), and “sprain injury” (RRR = 0.20, p = 0.007) at the baseline were significant factors. The findings indicate that previous fall experiences, higher alcohol consumption, lower frailty scores, and poor balance levels are associated with fall injury patterns. These patterns should be considered when developing prevention interventions.

## Introduction

Falls are the leading cause of unintentional injuries and death among older adults^1^. A 10-year follow-up study involving older adults who visited an emergency department with fall-induced injuries reported that falls are associated with the decline in their functional ability and increased mortality^2^. Specifically, the impacts of falls on older adults’ health differ depending on the types and levels of injuries. Among the older adult population, according to data obtained from the Centers for Disease Control and Prevention, USA^3^, one in five falls results in serious injuries (fractures and/or head injuries). Florence et al.^4^ determined that fatal falls among older adults result in substantial medical costs and underlined the need for an effective fall prevention strategy. Additionally, if the fracture sites are different, the risk factors and their associated adverse consequences are also different^5^. Therefore, when evaluating falls and their effects, it is necessary to consider not only the causes but also the fracture sites and associated factors.

Previous studies on the factors associated with falls have identified demographic (age, gender, educational level, economic status)^6,7^, behavioral (alcohol consumption, exercise)^8,9^, physical (postural balance, frailty)^10–12^, and environmental factors like home hazards^13^. According to previous studies’ results, the risks of falling are associated with advanced age^6,7^, being female^6,7^, lower economic and education levels^7^, high alcohol consumption level^8^, low physical activity level^11^, poor postural balance^9,11^, high frailty scores^10^, and various home hazards^13^. However, many previous studies on falls among older adults only focus on the fragmentary characteristics of falls, such as whether the individuals involved have experienced one fall or recurrent falls, as the dependent variables^10^. Therefore, such findings have limitations, as they pertain to the development of practical strategies that can be applied to prevent falls among older adults. A study investigating nurses’ perceptions on the implementation of fall prevention interventions also emphasized on the importance of providing tailored interventions that consider the patients’ fall risk factors^14^.

To suggest effective fall prevention strategies for older adults, it is necessary to derive fall patterns, while considering the characteristics of falls, and identify factors affecting fall patterns associated with serious injuries. Latent class analysis (LCA) is a representative analysis method that involves combining various characteristics to identify meaningful patterns in various health problems^15^. In previous studies, the patterns of various phenomena of interest, such as patterns of lifetime comorbidity among patients suffering from posttraumatic stress disorder^16^, categories of risk factors associated with suicide^17^, and patterns of adverse experiences during childhood^18^, were suggested using LCA analysis. Based on the derived patterns, various researchers have suggested high-severity patterns to which healthcare providers should pay attention^18^ and emphasized the need for differentiated interventions^17^. Therefore, combining the characteristics of various types of falls with the related injuries may help in identifying the patterns associated with serious injuries. Additionally, identifying the risk factors associated with serious fall injury patterns may provide basic data for the development of prevention strategies on which healthcare providers should prioritize and focus.

In this study, fall injury patterns and their associated factors were investigated using data obtained from the baseline (2016–2017; Time 1) and follow-up (2018–2019; Time 2) periods of the Korean Frailty and Aging Cohort Study (KFACS). The specific research objectives were as follows:

1. To identify fall injury patterns among community-dwelling older adults with fall experiences during the past year using the baseline and follow-up data obtained from the KFACS.
2. To identify the transition of fall injury patterns from the baseline to the follow-up period.
3. To identify various factors, such as fall latent classes at the baseline and frailty, associated with the identified fall injury patterns during the follow-up period.

## Methods

In this study, we performed a secondary analysis using data obtained from the KFACS, which is an ongoing, nationwide, population-based cohort study involving older adults in Korea^19^.

### Data source

The KFACS is a national cohort study involving community-dwelling older adults^19^. The purpose of this cohort study was to engage with participants to examine and analyze frailty in terms of its status and its associated factors as well as the transitions between different states of frailty^19^. The variables used in the cohort study mentioned above included demographics, health behaviors, health status, healthcare, social functions, cognitive functions, anthropometry, physical functions, health assessments, body composition, panoramic radiography, and the assessment of frailty and sarcopenia^19^. Baseline data collection was conducted by trained investigators at 10 centers nationwide, with follow-ups being conducted every two years^19^. The data are open to approved researchers^19^. Detailed information regarding the cohort profile has been presented in the work of Won et al.^19^.

### Sample

The baseline sample comprised 3014 community-dwelling older adults aged 70–84 years. The participants were selected using quota sampling, stratified by age and gender. This study used data obtained from two time points, which are currently available for research purposes. Among the initial 3014 participants, 2400 had no experience of falls at Time 1, and 48 participants provided missing data for the fall experience variable at Time 1 or Time 2. Therefore, these individuals were excluded from this study. In this study, the differences between the baseline characteristics of the excluded participants and those of the included participants have been presented in Supplementary Table 1. Such participants were excluded because we aimed to analyze the fall injury patterns of the participants who experienced falls and identify the transition and risk factors for recurrent falls. Therefore, the data involving 566 individuals with fall experience during the past year were included in the analysis process.

### Measures

#### Falls

In this study, using data obtained from the KFACS, we considered 11 items concerning falls among older adults to identify the fall injury patterns involved. The 11 items comprised the following three subgroups: (1) experience of falls over the previous 12 months (one item: “Have you fallen in the last 12 months?”), (2) injuries due to falls (four items: sprains; bruises or lacerations; fractures; and other), and (3) sites of fractures due to falls (six items: upper extremities, wrist, or hand; pelvis, hip, or femur; lower extremities, ankle or foot; spine; ribs or clavicle; and other). Each variable was coded as “1” if there was a previous fall experience, injury, or fracture site and “0” otherwise. Using these 11 items, we statistically derived the associated fall injury patterns through LCA analysis.

#### Frailty

In the KFACS, frailty is assessed using a modified version of the Fried frailty phenotype, which comprises five components: unintended weight loss, weakness, self-reported exhaustion, slowness, and low levels of physical activity^19–21^. Unintentional weight loss was evaluated using the following question: “In the last year, have you lost more than 4.5 kg unintentionally?” Weakness was measured using a grip strength test involving the use of both hands among the participants. The strength of each hand was tested twice using a hand grip dynamometer (T.K.K.5401, Takei Scientific Instruments Co, Tokyo, Japan). The confirmed maximum value was normalized based on gender, body mass index, and whether the value was within the lower 20th percentile of the sample^19^. Self-reported exhaustion was evaluated using the following items contained in the Center for Epidemiologic Studies-Depression Scale: “I felt that everything I did was an effort” and “I could not get going.” The respondents’ answers were based on whether they experienced the feeling in question for three or more days per week^19^. Slowness was evaluated by measuring the time taken to walk for 4 m, along with acceleration and deceleration phases of 1.5 m, using an automatic timer, and for those whose the “time taken” variable appeared in the lower 20th percentile of the sample data were classified as slow^19^. Low levels of physical activity was calculated using the International Physical Activity Questionnaire (IPAQ) to determine the participants’ kcal/week and metabolic equivalent (MET) scores, and it was defined as < 494.65 kcal for males and < 283.50 kcal for females^19^. This result corresponded to the lower 20th percentile observed in a population-based Korean survey focusing on the total energy consumed among older adults^19^. For each participant, the total frailty scores (range: 0–5) were summed by assigning one point for each of the following items: unintended weight loss of more than 4.5 kg, whether the participants answered “yes” in the self-report questionnaire regarding exhaustion, grip strength, 4-m walk time, and whether each of the activity levels were in the lower 20^th^ percentile^19^. Participants with a score of 3 or higher were classified as frail^19,20^.

#### Health-related and demographic factors at the baseline

The health-related factors measured at the baseline included the frequency of alcohol consumption, physical activity, and balance. The frequency of alcohol consumption was evaluated using the following item: “During the past year, how frequently did you consume alcohol?” The responses were categorized as follows: “I did not drink at all,” “between once a year and four times a month,” or “twice a week or more.” Physical activity was assessed using the IPAQ^22,23^. To derive total energy expenditure during physical activity, the MET scores were calculated according to the IPAQ scoring protocol (MET level × minutes of activity × events per week). Balance was measured by evaluating the number of seconds participants could maintain balance in the tandem position, and performance was categorized as “less than 3 s,” “3–9.9 s,” and “more than 10 s”^24^. In this study, by referring to previous studies, the demographic factors for which data were collected included age, gender, educational level, public assistant beneficiary status, marital status, residential area, and housing status^6,7,13^.

### Statistical analysis

The following statistical analysis methods and procedures were employed in this study. LCA is an analytical method that combines various categorical variables to identify latent classes and, consequently, it presents meaningful patterns^15,25^. Latent class prevalence and item-response probability indicate the probability of membership in each latent class and the probability of an observed response on a variable conditional on latent class membership^15^, respectively. Additionally, the item probabilities > 0.70 and < 0.30 mean that the derived latent class has high homogeneity levels^26^. Latent transition analysis (LTA) is used to model transitions of latent class membership derived through LCA over time through longitudinal data^15^. LCA was performed using SAS PROC LCA to identify latent classes (patterns) of falls for Time 1 and Time 2 by combining fall injury characteristics. Then, LTA was conducted using SAS PROC LTA to identify the transition probability of the fall injury patterns from Time 1 to Time 2, referring to changes in the identified patterns over time. The Akaike information criterion (AIC), the Bayesian information criterion (BIC), and entropy were used as fitness indices to determine the most appropriate number of potential patterns for each time point^15^. In the cases involving the use of the AIC and the BIC, smaller values indicated enhanced model suitability. In the case of entropy, values closer to 1 indicated enhanced model suitability. In the cases where the models recommended by the AIC and BIC differed, the researcher selected an appropriate number while considering parsimony or conceptual appeal^15^.

“Fall injury patterns” was identified as a dependent variable for Time 2. Chi-square tests and analyses of variance were conducted to confirm the differences between the fall injury patterns at Time 2. Multinomial logistic regression (MLR) was conducted to identify the factors associated with fall injury patterns at Time 2. The goodness-of-fit of MLR was tested using the Hosmer–Lemeshow goodness-of-fit test^27^. The results indicated that the fit of the model was adequate (χ^2^ = 13.723, df = 16, p-value = 0.619). SAS 9.4 (SAS Institute, Inc., Cary, NC) and SPSS 25.0 (IBM Corp., Armonk, N.Y., USA) were used to conduct the analyses mentioned above.

### Ethics approval and consent to participate

This study was approved by the Institutional Review Board of Kyung Hee University Medical Center (IRB no. KHUH-2021-02-037). Written informed consent was obtained from all the participants. All the methods used in this study were used in accordance with the guidelines and regulations of the Declaration of Helsinki.

## Results

### Participant characteristics

Table 1 shows the baseline characteristics of the participants. The mean age of the participants was 76.31 (SD = 3.75). Out of 566 participants, 358 participants were female, and the mean frailty score of the participants was 1.15 (SD = 1.16).

**Table 1:** Participants’ baseline characteristics (N = 566).

Variable	Category	n (%) or Mean ± SD
Age (years)		76.31 ± 3.75
Gender	Male	208 (36.7)
	Female	358 (63.3)
Educational level	Elementary school	319 (56.5)
	Middle school	77 (13.6)
	High school	98 (17.3)
	College	71 (12.6)
Public assistant beneficiary	Yes	55 (9.8)
	No	507 (90.2)
Marital status	Married	320 (56.6)
	Bereaved	226 (39.9)
	Other	20 (3.5)
Area of residence	Urban	147 (26.1)
	Suburban	252 (44.7)
	Rural	165 (29.2)
Housing status	Detached house	250 (44.2)
	Apartment	230 (40.6)
	Other	86 (15.2)
Drinking	Not drinking	316 (56.2)
	1/year–4/month	166 (29.6)
	≥ 2 times/week	80 (14.2)
Physical activity (MET-min/week)		2652.95 ± 3416.72
Balance (tandem position)	< 3 s	69 (12.5)
	3–9.9 s	76 (13.8)
	≥ 10 s	407 (73.7)
Frailty (score)		1.15 ± 1.16

SD, standard deviation; MET, metabolic equivalent.

Table 2 shows the fall injury characteristics of the participants at Time 1 and Time 2. At Time 1, among the 566 participants who experienced falls, 61 had sprains, 276 had bruises or lacerations, and 92 had fractures. Among the 566 participants who experienced falls at Time 1, 220 experienced falls again at Time 2. Among the 220 participants, 22 had sprains, 102 had bruises or lacerations, and 26 had fractures.

**Table 2:** Fall injury characteristics of participants (N = 566).

Variable	Category	Time 1		Time 2	
		N	%	n	%
Fall experience	Yes	566	100.0	220	38.9
	No	0	0.0	346	61.1
Injuries from fall: sprain	Yes	61	10.8	22	3.9
	No	505	89.2	544	96.1
Injuries from fall: bruises, lacerations	Yes	276	48.8	102	18.0
	No	290	51.2	464	82.0
Injuries from fall: fractures	Yes	92	16.3	26	4.6
	No	474	83.7	540	95.4
Injuries from fall: others	Yes	21	3.7	7	1.2
	No	545	96.3	559	98.8
Fracture site: upper extremity, wrist, hand	Yes	22	3.9	7	1.2
	No	544	96.1	559	98.8
Fracture site: pelvis, hip, femur	Yes	16	2.8	3	0.5
	No	550	97.2	563	99.5
Fracture site: lower extremity, ankle, foot	Yes	23	4.1	3	0.5
	No	543	95.9	563	99.5
Fracture site: spine	Yes	12	2.1	7	1.2
	No	554	97.9	559	98.8
Fracture site: rib, clavicle	Yes	20	3.5	4	0.7
	No	546	96.5	562	99.3
Fracture site: other	Yes	5	0.9	1	0.2
	No	561	99.1	565	99.8

### Fall injury patterns and transition

Table 3 shows the results of the analysis of each of the fall injury patterns, which were determined through LCA, at Time 1 and Time 2. It also shows the transition probability, which was determined using the LTA of the patterns from Time 1 to Time 2. At Time 1, the following three patterns were identified: (1) bruising injuries (46.5%), (2) sprain injuries (37.3%), and (3) fracture injuries (16.2%). At Time 2, there were three patterns: (1) no fall (61.1%), (2) bruising and/or sprain injuries (34.3%), and (3) fracture injuries (4.6%). When applying LCA to identify fall injury patterns, we established that the BIC levels increased when the number of patterns increased from three to four (Table 4). However, the AIC scores continued to decrease, even when the number of patterns increased to four at Time 1. Therefore, the researchers decided that the best-fitting number of patterns was three, considering the parsimony of the results of the analysis^15^.

**Table 3:** Results of the latent class analysis and latent transition analysis of fall injury characteristics.

Item-response probabilities	Latent class		
	Bruising injury (n = 263, 46.5%)	Sprain injury (n = 211, 37.3%)	Fracture injury (n = 92, 16.2%)
**Time 1**			
Fall experience	**1.0000**	**1.0000**	**1.0000**
Injuries from fall: sprains	0.0304	0.2322	0.0435
Injuries from fall: bruises, lacerations	**1.0000**	0.0000	0.1413
Injuries from fall: fractures	0.0000	0.0000	**1.0000**
Injuries from fall: other	0.0000	0.0948	0.0109
Fracture site: upper extremity, wrist, hand	0.0000	0.0000	0.2391
Fracture site: pelvis, hip, femur	0.0000	0.0000	0.1739
Fracture site: lower extremity, ankle, foot	0.0000	0.0000	0.2500
Fracture site: spine	0.0000	0.0000	0.1304
Fracture site: rib, clavicle	0.0000	0.0000	0.2174
Fracture site: other	0.0000	0.0000	0.0543

Item-response probabilities	Latent class		
	No fall (n = 346, 61.1%)	Bruising and/or sprain injury (n = 194, 34.3%)	Fracture injury (n = 26, 4.6%)
**Time 2**			
Fall experience	0.0000	**1.0000**	**1.0000**
Injuries from fall: sprains	0.0000	0.0979	0.1154
Injuries from fall: bruises, lacerations	0.0000	**0.5155**	0.0769
Injuries from fall: fractures	0.0000	0.0000	**1.0000**
Injuries from fall: other	0.0000	0.0309	0.0385
Fracture site: upper extremity, wrist, hand	0.0000	0.0000	0.2692
Fracture site: pelvis, hip, femur	0.0000	0.0000	0.1154
Fracture site: lower extremity, ankle, foot	0.0000	0.0000	0.1154
Fracture site: spine	0.0000	0.0000	0.2692
Fracture site: rib, clavicle	0.0000	0.0000	0.1538
Fracture site: other	0.0000	0.0000	0.0385

Probability of transition			
	Time 2 latent status		
Time 1 latent status	No fall	Bruising and/or sprain injury	Fracture injury
Bruising injury	**0.5741**	0.3916	0.0342
Sprain injury	**0.6635**	0.2986	0.0379
Fracture injury	**0.5978**	0.3043	0.0978

Item response probabilities of > 0.5 are represented in bold.

**Table 4:** Model fit and percentage of participants in each latent class.

No. of Classes	AIC	BIC	Entropy	Percentage of participants in each class			
				Class 1	Class 2	Class 3	Class 4
**Time 1**							
2	293.90	393.69	1.00	0.8375	0.1625		
3	236.44	388.29	1.00	0.4647	0.3728	0.1625	
4	222.00	425.92	1.00	0.4647	0.3728	0.1272	0.0353
**Time 2**							
2	255.87	355.65	1.00	0.6113	0.3887		
3	147.05	298.90	1.00	0.6113	0.3428	0.0459	
4	159.27	363.18	0.39	0.4962	0.1933	0.1853	0.1252

AIC, akaike information criterion; BIC, Bayesian information criterion.

For all the latent statuses for Time 1, the probability of experiencing another fall with minor injuries, such as bruising and lacerations, at Time 2 was greater than 0.29. Additionally, for the latent status of experiencing a fall resulting in a severe injury (e.g., a fracture) at Time 1, the probability of re-experiencing a severe fall-induced injury at Time 2 was the highest, at 0.098. When applying LTA to identify transition probability, we established that the BIC scores increased when the number of latent statuses increased from three to four (Supplementary Table 2). Therefore, the researchers decided that the number of latent statuses was three.

### Association between the baseline characteristics and fall injury patterns at Time 2

Table 5 shows the differences among Time 2 patterns regarding demographic and health-related characteristics. There were statistically significant differences between the patterns regarding gender (χ^2^ = 8.56, p = 0.014), educational level (χ^2^ = 13.08, p = 0.042), balance (in the tandem position) (χ^2^ = 18.81, p = 0.001), fall injury patterns at Time 1 (χ^2^ = 11.47, p = 0.022), and frailty (F = 5.27, p = 0.005).

**Table 5:** Relationships between baseline characteristics and fall injury patterns at Time 2.

Variable	Category	n (%) or Mean ± SD				χ^2^ or F	*p*
		Total (N = 566)	No fall (n = 346)	Bruising and/or sprain injury (n = 194)	Fracture injury (n = 26)		
Age (years)	–	76.31 ± 3.75	76.05 ± 3.74	76.72 ± 3.78	76.62 ± 3.50	2.04	0.131
Gender	Male	208 (36.7)	137 (39.6)	68 (35.1)	3 (11.5)	8.56	0.014
	Female	358 (63.3)	209 (60.4)	126 (64.9)	23 (88.5)		
Educational level	Elementary school	319 (56.5)	186 (53.9)	113 (58.2)	20 (76.9)	13.08	0.042
	Middle school	77 (13.6)	58 (16.8)	16 (8.2)	3 (11.5)		
	High school	98 (17.3)	58 (16.8)	38 (19.6)	2 (7.7)		
	College	71 (12.6)	43 (12.5)	27 (13.9)	1 (3.8)		
Public assistant beneficiary	Yes	55 (9.8)	37 (10.7)	14 (7.3)	4 (15.4)	2.57	0.276
	No	507 (90.2)	308 (89.3)	177 (92.7)	22 (84.6)		
Marital status	Married	320 (56.5)	201 (58.1)	107 (55.2)	12 (46.2)	3.30	0.509
	Bereaved	226 (39.9)	132 (38.2)	80 (41.2)	14 (53.8)		
	Other	20 (3.5)	13 (3.8)	7 (3.6)	0 (0.0)		
Area of residence	Urban	147 (26.1)	92 (26.7)	50 (25.8)	5 (19.2)	5.13	0.274
	Suburban	252 (44.7)	160 (46.5)	83 (42.8)	9 (34.6)		
	Rural	165 (29.3)	92 (26.7)	61 (31.4)	12 (46.2)		
Housing status	Detached house	250 (44.2)	141 (40.8)	94 (48.5)	15 (57.7)	5.58	0.233
	Apartment	230 (40.6)	152 (43.9)	70 (36.1)	8 (30.8)		
	Other	86 (15.2)	53 (15.3)	30 (15.5)	3 (11.5)		
Drinking	Not drinking	316 (56.2)	190 (55.2)	108 (56.3)	18 (69.2)	5.21	0.267
	1/year–4/month	166 (29.5)	108 (31.4)	51 (26.6)	7 (26.9)		
	≥ 2 times/week	80 (14.2)	46 (13.4)	33 (17.2)	1 (3.8)		
Physical activity (MET-min/week)	–	2652.95 ± 3416.72	2754.74 ± 3676.35	2405.22 ± 2902.46	3141.23 ± 3370.72	2.90^a^	0.234
Balance (tandem position)	< 3 s	69 (12.5)	34 (10.0)	29 (15.6)	6 (23.1)	18.81	0.001
	3–9.9 s	76 (13.8)	35 (10.3)	38 (20.4)	3 (11.5)		
	≥ 10 s	407 (73.7)	271 (79.7)	119 (64.0)	17 (65.4)		
Fall injury patterns at Time 1	Bruising injury	263 (46.5)	151 (43.6)	103 (53.1)	9 (34.6)	11.47	0.022
	Sprain injury	211 (37.3)	140 (40.5)	63 (32.5)	8 (30.8)		
	Fracture injury	92 (16.3)	55 (15.9)	28 (14.4)	9 (34.6)		
Frailty (score)	–	1.15 ± 1.16	1.04 ± 1.12	1.36 ± 1.21	0.87 ± 1.06	5.27	0.005

SD, standard deviation; MET, metabolic equivalent.

^a^Kruskal–Wallis test was performed.

### Factors associated with fall injury patterns at Time 2

The results of the MLR analysis are listed in Table 6. At Time 2, when using “no fall” as a reference, the factors associated with “bruising and/or sprain injury” were associated with middle school or below education levels compared to high school or above (relative risk ratio [RRR] = 0.55, p = 0.012), alcohol consumption between once a year and four times a month compared to drinking alcohol twice a week and higher (RRR = 0.50, p = 0.034), and the balance group of “3–9.9 s” in the tandem position compared to “more than 10 s” (RRR = 2.77, p < 0.001). The risk of being included in the “bruising and/or sprain injury” pattern was high if the participant had a higher educational level, increased levels of alcohol consumption, and poor balance.

**Table 6:** Results of multinomial logistic regression analysis examining risk factors for fall injury patterns at Time 2.

Variable	Category	Bruising and/or Sprain injury		Fracture injury	
		RRR	*p*	RRR	*p*
Age	–	1.02	0.575	1.11	0.125
Gender (ref. female)	Male	0.69	0.150	0.22	0.038
Education (ref. ≥ high school)	≤ Middle school	0.55	0.012	1.81	0.391
Public assistant beneficiary (ref. no)	Yes	0.83	0.622	1.46	0.598
Marital status (ref. other)	Married	1.01	0.955	1.80	0.245
Area (ref. rural)	Urban/suburban	0.78	0.307	0.39	0.073
Housing (ref. other)	Detached house	1.24	0.487	1.27	0.741
	Apartment	0.76	0.363	0.64	0.557
Drinking (ref. ≥ 2 times/week)	Not drinking	0.57	0.075	1.36	0.789
	1/year–4/month	0.50	0.034	0.79	0.847
Physical activity	–	1.00	0.609	1.00	0.874
Balance (ref. ≥ 10 s)	< 3 s	1.52	0.178	1.81	0.372
	3–9.9 s	2.77	< 0.001	1.35	0.679
Fall injury patterns at Time 1 (ref. fracture injury)	Bruising injury	1.16	0.616	0.23	0.007
	Sprain injury	0.69	0.233	0.20	0.007
Frailty	–	1.17	0.094	0.58	0.042

RRR, relative risk ratio; CI, confidence interval.

For Time 2, when using “no fall” as a reference, the factors associated with “fracture injury” were being male (RRR = 0.22, p = 0.038), frailty score (RRR = 0.58, p = 0.042), “bruising injury” patterns (RRR = 0.23, p = 0.007) and “sprain injury” patterns (RRR = 0.20, p = 0.007) compared to the “fracture injury” pattern at the baseline. The risk of being included in the “fracture injury” pattern was high if the participant was female, had a low frailty score, and had been in the “fracture injury” pattern at the baseline.

## Discussion

In this study, the fall injury patterns of community-dwelling older adults in Korea were identified, and the transition of these patterns over time was investigated. Additionally, by identifying the risk factors related to the fall injury patterns associated with high-risk injuries, such as fractures, this study’s findings highlight the aspects to be considered when assessing, planning, and designing interventions aimed at preventing falls among older adults.

Fall injury patterns were identified by combining various characteristics related to falls. At Time 2, three fall injury patterns were identified: no falls, bruising and/or sprain injuries, and fracture injuries. In previous studies, only the fragmentary characteristics of falls, such as previous experiences of falls or experiencing recurrent falls, were used as dependent variables^10^. However, falls are variables with diverse characteristics (e.g., cause, time, season, environment, and injury) that are difficult to explain simply by considering the presence or absence of a fall history^28,29^. Additionally, their impact on health and medical costs^4^ as well as the risk factors for falls may differ depending on the seriousness of the injuries^29^. Therefore, by identifying fall injury patterns through the analysis of related injuries and the associated factors, this study plays a significant role in presenting practical knowledge that may be useful for fall prevention among older adults. Additionally, to evaluate the validity of the LCA results, it is necessary to establish whether the number and characteristics of the derived classes are similar, even when the LCA is conducted using an external dataset^30^. Therefore, further studies should be conducted to confirm the validity of the results achieved through this study.

At Time 2, the rate of fracture injuries resulting from recurrent falls was high. Among the 566 older adults who experienced a fall at Time 1, the proportion of participants classified as “no fall” at Time 2 was the highest, at 61.1%. However, at Time 1, for the group with severe injuries, such as fractures, the probability of being part of the group with a “fracture injury” at Time 2 (i.e., experiencing another fall that resulted in a fracture) was 9.8%. Additionally, according to the results of the MLR analysis, the possibility of experiencing severe injuries, such as fractures at Time 1, was highly probable in the group with similar patterns at Time 2. Previous studies suggest that a history of falls is a risk factor for further falls^31,32^. Fall-related hospitalizations of older adults in New York City showed that 15% of the entire sample was hospitalized twice or more, and the average length of time between the first and second hospitalizations was approximately a year and a half (556 days)^33^. According to a multifactorial strategy aimed at the prevention of serious fall injuries among community-dwelling older adults, seven risk factors were included: the impairment of strength, gait, or balance; osteoporosis or vitamin D deficiency; vision impairment; problems with feet or footwear; use of fall-risk-increasing medication; postural hypotension; and home safety hazards^34^. As a result of evaluating the aforementioned factors through a randomized trial, the development of a multifactorial strategy that includes the assessment of the related risk factors, the development of an individualized prevention plan, and referral to a community-based program was effective in reducing the rates of fall-related injuries^34^. Therefore, annual systematic fall risk assessments, including physical and functional examinations, clinical assessments, and home assessments of individuals at a high risk of falls and fractures, along with the linkage of such assessments associated with the implementation of fall prevention interventions are necessary to avert serious fall injuries and recurrent falls^35^.

In Time 2, as a result of analyzing the differences among the three fall patterns, the modifiable factor that showed a statistically significant difference among participants was balancing. Additionally, the risk of experiencing falls that result in minor injuries, such as bruising and sprains, was higher in the group with poor balance. This result is consistent with the results of a previous study that reported poor balance being a prominent risk factor for falls^11,29,36^. Therefore, to prevent falls, it is important to assess balance among older adults and, if necessary, implement measures for improvement. In a review conducted by Roeing et al.^37^, mobile applications, accelerometers, force platforms, and three-dimension (3D) motion capture technological approaches were suggested as tools for assessing balance and fall risk. Further, as a representative intervention, step training (i.e., training in taking correct, rapid, and well-directed steps when walking) has been verified as an effective approach aimed at preventing falls among older adults and strengthening their balance^38^. Therefore, providing education aimed at increasing the levels of awareness and engagement in intervention measures and fostering collaboration among healthcare providers, physical therapists, and occupational therapists^14^ in enhancing balance and preventing falls among older adults should be considered.

In this study, high frailty scores were determined to be associated with decreased risks of fracture-related injuries. This finding differs from that of a previous meta-analysis in which frailty was reported as a risk factor for falls among community-dwelling older adults^10^. Contrarily, a previous study that analyzed the relationship between gait speed and falls suggested that the risk of outdoor falls was significantly high in the group with faster gait speed^39^. Therefore, in this study, it can be inferred that most of the participants may have an increased risk of falls because of increased outdoor activity compared to the frail participants. However, the findings of this study may be accounted to the presence of a small proportion of frail individuals in the sample. The KFACS included older adults who could visit a medical center at baseline assessment, and only 7.8% of these individuals were frail^19^. In other words, a possible limitation to this study is its sample of a relatively healthy population. Additionally, only the baseline data and two years’ follow-up data were used in this study. Therefore, it is necessary to re-evaluate the association between frailty and falls involving a longer follow-up period.

The risk of experiencing a fall resulting in minor injuries was higher among the group that consumed alcohol heavily (twice a week or more). This result is consistent with the results of previous studies. One study reported that alcohol consumption is a statistically significant risk factor contributing to falls and associated injuries^8^. According to a study conducted by Hwang and Kim^40^, the researchers reviewed the records of 2,092 older adults who visited the emergency department as a result of alcohol-related falls, and they found that 91% of patients did not have severe injuries and 80.8% of them were discharged without admission. Additionally, according to a study on the risk factors for falling, by dividing falls into indoor falls and outdoor falls, the authors argued that high or moderately high alcohol consumption levels (one drink a week or more) were risk factors for outdoor falls^41^. Because alcohol consumption is a modifiable risk factor for falls, one potential strategy for preventing falls among older adults may be the modification of alcohol-consumption behavior^42^. Therefore, when establishing a strategy for preventing falls that result in minor injuries among older adults, alcohol consumption should be considered, and appropriate interventions aimed at raising awareness of the risk of falls and strengthening education on the prevention of falls associated with high-risk levels of alcohol consumption should also be considered.

According to this study, females are at an increased risk of serious injuries, such as fractures, resulting from falls. According to a cross-sectional study on Japanese community-dwelling adults aged 40 and older, the risk of falls resulting in fractures was 2.99 times higher among females^43^. This result needs to be considered together with the increased risk of osteoporosis and osteopenia among females. Among females, bone loss begins earlier compared to the same among males. Among females aged over 50 years, the rate of osteoporosis and osteopenia has been reported to be four times and two times higher than the same among their male peers, respectively^44^. Previous studies have also noted gender-based differences, as they pertain to fall risk factors. One study reported that living alone, the dependency on any instrumental activity of daily living, being underweight, having a cognitive impairment, and taking fall risk-increasing drugs are related to injurious falls among females but not males^31^. Additionally, compared to males, females are highly likely to fall because of trips or stumbles when walking^45^. Therefore, females are at an increased risk of falls that cause serious injuries. Additionally, because the risk factors for falls differ by gender^46^, preventive intervention planners should consider gender differences along with other risk factors associated with falls that result in serious injuries.

The risk of falls resulting in minor injuries was higher in the group with a high educational level. This finding differs from the results of a previous study arguing that low educational level is a significant risk factor for falls among older adults^7^. However, several studies have reported that a high educational level is significantly related to a high level of physical activity^47^. Following the further analysis of educational level and physical activity by dividing them into three categories (low, moderate, or higher), the rate of moderate or increased physical activity was statistically significantly higher in the group with a high educational level compared to the group with a low educational level (84.6% vs. 76.3%, χ^2^ = 4.94, p = 0.026). It can be inferred that the current findings related to the association between high educational levels and minor fall-induced injuries could be because of a higher degree of physical activity among people with a higher educational level, which may result in falls. However, because the data used in this study did not include information regarding activities that result in falls, additional investigation is required.

This study has the following limitations. First, the findings were derived from secondary data analysis, and the available variables that could be included in the analysis were limited. A re-analysis involving additional information related to specific falls (time when the fall occurred, classification of indoor/outdoor falls, location where the fall occurred, cause of the fall, number of falls, and injurious falls, etc.) as well as medical conditions, such as osteoporosis treatment status and medication, that may affect the occurrence of falls is necessary future studies. Second, in this study, only data obtained from two time points (the baseline and the first follow-up period) were used for analysis. In future studies, a re-analysis will be necessary using additionally collected long-term data. Third, the question regarding “experience of falls during the previous 12 months” was asked while examining fall experiences, thereby resulting in limited information regarding the fall injury patterns over the past two years. Additionally, according to a previous study, falls must be measured once a month to prevent recall bias^48^. Therefore, in this study, recall bias may have affected the results of the survey on fall experience. Fourth, the number of participants who experienced fracture injury due to falls was small. Therefore, additional studies involving a sufficient sample size are required in future studies. Finally, the generalizability of the results is limited because the participants were relatively healthy.

## Conclusions

In this study, we analyzed longitudinal data relating to falls among community-dwelling older adults in Korea, and we identified fall injury patterns and the associated factors throughout the two-year follow-up process. Through LCA analysis, three fall injury patterns (a group that did not experience falls, a group that experienced bruises and lacerations, and a group that experienced fractures) were identified. Factors associated with falls resulting in bruises and/or sprains throughout the two-year follow-up period were high education level, high levels of alcohol consumption, and poor balance. Meanwhile, factors associated with falls resulting in fractures during the two-year follow-up period were being female, having low frailty, and a history of fall-induced fractures. The findings of this study suggest that regular fall risk screening and interventions designed for community-dwelling older adults is crucial. Furthermore, healthcare providers should provide individualized, effective, and available fall prevention interventions based on a fall risk assessment, including fall history, physical examination, and modifiable health risk behaviors.

## Supplementary Information

Supplementary Tables.

## Data Availability

The data that support the findings of this study are available from Korean Frailty and Aging Cohort Study (KFACS). Restrictions apply to the availability of these data, which were used under license for this study. Data are available from the corresponding author, MS, with the permission of KFACS.
