# Supplementary material for: Patterns and predictors of fall injury transitions among Korean older adult fallers: a 2-year longitudinal study
Source: Sci Rep. 2022 Dec 23;12:22188. doi: 10.1038/s41598-022-26665-2 (PMC9789049; doi:10.1038/s41598-022-26665-2)
Supplement: Supplementary file 1 — Supplementary Tables. [file 41598_2022_26665_MOESM1_ESM.docx]

Table S1. Differences of baseline characteristics between included and excluded participants according to the missing fall experience variable (N=614)

| **Variable** | **Category** | **n (%) or Mean±SD** | | | **χ^2^ or t** | ***p*** |
| --- | --- | --- | --- | --- | --- | --- |
|  |  | **Total**  **(N=614)** | **Included**  **(n=566)** | **Excluded (n=48)** |  |  |
| **Age (years)** | **-** | 76.37±3.74 | 76.31±3.75 | 77.06±3.66 | 1.343 | .180 |
| **Gender** | **Male** | 229 (37.3) | 208 (36.7) | 21 (43.8) | 0.927 | .336 |
|  | **Female** | 358 (62.7) | 358 (63.3) | 27 (56.3) |  |  |
| **Educational level** | **≤Middle school** | 427 (69.7) | 396 (70.1) | 31 (64.6) | 0.634 | .426 |
|  | **≥High school** | 186 (30.3) | 169 (29.9) | 17 (35.4) |  |  |
| **Public assistant beneficiary** | **Yes** | 59 (9.7) | 55 (9.8) | 4 (8.5) | 0.081 | .811 |
|  | **No** | 550 (90.3) | 507 (90.2) | 43 (91.5) |  |  |
| **Marital status** | **Married** | 349 (56.8) | 320 (56.5) | 29 (60.4) | 0.271 | .602 |
|  | **Others^a^** | 265 (43.2) | 246 (43.5) | 19 (39.6) |  |  |
| **Area of residence** | **Urban/suburban** | 426 (69.7) | 399 (70.7) | 27 (57.4) | 3.634 | .057 |
|  | **Rural** | 185 (30.3) | 165 (29.3) | 20 (42.6) |  |  |
| **Housing status** | **Detached house** | 279 (45.4) | 250 (44.2) | 29 (60.4) | 10.799 | .005 |
|  | **Apartment** | 238 (38.8) | 230 (40.6) | 8 (16.7) |  |  |
|  | **Others** | 97 (15.8) | 86 (15.2) | 11 (22.9) |  |  |
| **Drinking** | **Not drinking** | 344 (56.4) | 316 (56.2) | 28 (59.6) | 4.792 | .091 |
|  | **1/year–4/month** | 174 (28.6) | 166 (29.5) | 8 (17.0) |  |  |
|  | ≥2 **times/week** | 91 (14.9) | 80 (14.2) | 11 (23.4) |  |  |
| **Physical activity (MET-min/week)** | - | 2618.62± 3330.98 | 2652.95± 3416.72 | 2197.71± 1976.74 | 0.891 | .373 |
| **Balance (tandem position)** | **<3 sec** | 438 (73.1) | 407 (73.7) | 31 (66.0) | 1.437 | .487 |
|  | **3–9.9 sec** | 85 (14.2) | 76 (13.8) | 9 (19.1) |  |  |
|  | ≥**10 sec** | 76 (12.7) | 69 (12.5) | 7 (14.9) |  |  |
| **Frailty (score)** | **-** | 1.16±1.15 | 1.15±1.16 | 1.34±1.08 | 1.077 | .282 |

Note*.* SD = standard deviation; MET = metabolic equivalent

a. bereaved, separated, and divorced
Non-responses were excluded from the analysis.

Table S2. Model fit and percentage of participants in each latent status

| **No. of  latent statuses** | **G^2^** | **AIC** | **BIC** | **Percentage of participants in each class** | |
| --- | --- | --- | --- | --- | --- |
|  |  |  |  | **Wave** | **Percentage of participants** |
| 2 | 634.58 | 728.58 | 932.50 | 1 | Class 1: 83.8, Class 2: 16.3 |
|  |  |  |  | 2 | Class 1: 61.1, Class 2: 38.9 |
| 3 | 410.19 | 558.19 | 879.25 | 1 | Class 1: 46.5, Class 2: 37.3, Class 3: 16.2 |
|  |  |  |  | 2 | Class 1: 61.1, Class 2: 34.3, Class 3: 4.6 |
| 4 | 341.01 | 547.01 | 993.89 | 1 | Class 1: 46.5, Class 2: 37.3, Class 3: 12.7, Class 4: 3.5 |
|  |  |  |  | 2 | Class 1: 61.1, Class 2: 17.7, Class 3: 16.6, Class 4: 4.6 |

Note*.* AIC = Akaike information criterion; BIC = Bayesian information criterion
